# Value Addition in the Efficacy of Conventional Antibiotics by Nisin against *Salmonella*

**DOI:** 10.1371/journal.pone.0076844

**Published:** 2013-10-08

**Authors:** Aman Preet Singh, Vijay Prabha, Praveen Rishi

**Affiliations:** Department of Microbiology, Basic Medical Sciences Block, Panjab University, Chandigarh, India; Wadsworth Center, New York State Dept. Health, United States of America

## Abstract

Frequent and indiscriminate use of existing battery of antibiotics has led to the development of multi drug resistant (MDR) strains of pathogens. As decreasing the concentration of the antibiotic required to treat Salmonellosis might help in combating the development of resistant strains, the present study was designed to assess the synergistic effects, if any, of nisin, in combination with conventional anti-*Salmonella* antibiotics against *Salmonella enterica* serovar Typhimurium. Minimum inhibitory concentrations (MICs) of the selected antimicrobial agents were determined by micro and macro broth dilution assays. *In-vitro* synergy between the agents was evaluated by radial diffusion assay, fractional inhibitory concentration (FIC) index (checkerboard test) and time-kill assay. Scanning electron microscopy (SEM) was also performed to substantiate the effect of the combinations. *In-vivo* synergistic efficacy of the combinations selected on the basis of *in-vitro* results was also evaluated in the murine model, in terms of reduction in the number of Salmonellae in liver, spleen and intestine. Nisin-ampicillin and nisin-EDTA combinations were observed to have additive effects, whereas the combinations of nisin-ceftriaxone and nisin-cefotaxime were found to be highly synergistic against serovar Typhimurium as evident by checkerboard test and time-kill assay. SEM results revealed marked changes on the outer membrane of the bacterial cells treated with various combinations. *In-vivo* synergy was evident from the larger log unit decreases in all the target organs of mice treated with the combinations than in those treated with drugs alone. This study thus highlights that nisin has the potential to act in conjunction with conventional antibiotics at much lower MICs. These observations seem to be significant, as reducing the therapeutic concentrations of antibiotics may be a valuable strategy for avoiding/reducing the development of emerging antibiotic resistance. Value added potential of nisin in the efficacy of conventional antibiotics may thus be exploited not only against *Salmonella* but against other Gram-negative infections as well.

## Introduction

Most of the gastrointestinal tract infections, except those resulting in systemic disease such as typhoid fever, may not be treated with antibiotics unless affecting patients with underlying illnesses and having complicated febrile or immuno-compromised state [1]. However, the general trend has been the use of antibiotics to treat even mild gastroenteritis caused by non-typhoidal *Salmonella* [2], [3]. Thus frequent and indiscriminate use has led to the development of pathogenic bacteria that are resistant to the existing battery of antibiotics [4], [5]. The efficacy of these drugs has now been questioned with the emergence of resistance to traditional first line antibiotics as well as increased MICs of second generation quinolones [5]. This problem has prompted efforts towards the development of effective alternatives with limited avenues for bacterial drug resistance. Therefore, to combat such infections and to reduce the chances of emergence of resistance, co-therapy using two or more antimicrobial agents is being exploited [6]–[8].

In this context, antimicrobial peptides from prokaryotic and eukaryotic origin have attracted much attention due to their favorable properties in contrast to conventional antibiotics [9]–[11]. One promising alternative is the use of nisin, a ribosomally synthesized and post-translationally modified bacteriocin (Class I bacteriocin) produced by *Lactococcus lactis* subsp. *lactis*. This peptide from prokaryotic origin has been in use for decades in the food industry and has not been reported to induce widespread resistance. It is the only bacteriocin approved as “Generally Regarded As Safe” (GRAS) compound for use as food preservative in over 50 countries [12]. Nisin, though generally successful against Gram-positive organisms, has been found to be effective against Gram-negative organisms only in the presence of EDTA [13], [14] and some other agents like citrate [15], lysozyme [16] and plant essential oils [17], [18]. Its use in clinical trials is restricted due to the limitations on *in-vivo* use of EDTA. It has been suggested that the sensitization of Gram-negative organisms by chelating agents may not work since the concomitant acid production can antagonize the chelation reaction [19].

Recently, it has been revealed that antimicrobial peptides combined with clinically used antibiotics could be the alternatives to solve the problem of antibiotic-resistance [7], [20]. The present study was therefore, aimed at evaluating the potential of nisin to increase the efficacy of conventional antibiotics coupled with the possibility of preventing or at least reducing the chances of emergence of resistance in *Salmonella enterica* serovar Typhimurium. To the best of our knowledge, no information has been available on the combined activity of nisin and conventional anti-*Salmonella* drugs.

## Materials and Methods

### Ethics statement

The experimental protocols were approved by the Institutional Animal Ethics Committee (Approval ID: IAEC/156 dated 25.08.2011) of the Panjab University, Chandigarh, India (Registration number: 45/1999/CPCSEA) and performed in accordance with the guidelines of Committee for the Purpose of Control and Supervision of Experiments on Animals (CPCSEA), Government of India, on animal experimentation. All the efforts were made to minimize the suffering of animals.

### Animals

Female BALB/c mice (18 to 22 g, 4 to 5 weeks old) were procured from Central Animal House, Panjab University, Chandigarh (India). The animals were housed under standard conditions with free access to food and water *ad libitum*.

### Bacterial strain and growth medium

Standard strain of *Salmonella enterica* serovar Typhimurium NCTC74, originally provided by Central Research Institute, Kasauli, India, was used in the present study. This strain has been maintained in our laboratory for the last several years and has also been used in recent studies. For the sake of standardization in the pilot studies, the strain was maintained on MacConkey agar plates at 4°C and subcultured every two weeks on the same medium as well as on nutrient agar slants (37°C). Thereafter, every time glycerol stocks were used. Stock cultures were stored at −80°C in glycerol (20%). Purity of the strain was confirmed biochemically as well as serologically.

For preparation of bacterial cell suspension, bacterial cells grown overnight (at 37°C, 150 rpm) in nutrient broth (5.0 g/liter peptone, 5.0 g/liter NaCl, 1.5 g/liter beef extract, 1.5 g/liter yeast extract, pH 7.4±0.2) were harvested by centrifugation (8000 rpm, 15 min), washed once with 10 mM sodium phosphate-buffered saline (PBS, pH 7.2), and resuspended in PBS to a final concentration of approximately 10^7^ CFU/ml.

### Agents

Nisin, EDTA, ampicillin (ampi), chloramphenicol (chlor), ciprofloxacin (cipro), ceftriaxone (cef) and cefotaxime (cefo) powder were obtained from Sigma Aldrich (St Louis, MO, USA). Ampicillin, chloramphenicol, ceftriaxone, cefotaxime and EDTA were dissolved in distilled water whereas, ciprofloxacin and nisin were dissolved in 0.1 N HCl and 0.2N HCl respectively. Stock solutions of 500 mM for EDTA and 1 mg/ml for other agents were prepared and used within one week.

### Radial diffusion assay

The feasibility of using nisin in combination with ampicillin, chloramphenicol, ciprofloxacin, ceftriaxone, cefotaxime and EDTA was confirmed by the radial diffusion assay as described by us earlier [21]. Following the addition of control sample (normal saline) and test agents alone in each well, combinations of nisin-EDTA, nisin-ampicillin, nisin-chloramphenicol, nisin-ciprofloxacin, nisin-ceftriaxone and nisin-cefotaxime were added separately in wells. The plates were then incubated at 37°C for 24 hours and observed for the zones of inhibition around the wells. Anti-bacterial activity was evaluated by measuring the size of the clear zone of inhibition of bacterial growth around the wells.

### Quantitative determination of the antibacterial activity (MIC determination)

On the basis of radial diffusion assay, three antibiotics (ampicillin, ceftriaxone and cefotaxime) in addition to nisin and EDTA were chosen for further studies. The minimum inhibitory concentrations (MICs) of these agents against serovar Typhimurium were determined by macro and micro broth dilution assay methods. The MIC was defined as the lowest concentration of each antibiotic not producing any visible microbial growth.

#### (A) Macro broth dilution assay

Macro broth dilution assay was performed as described by us earlier [7]. In brief, cells were grown individually in the presence of different concentrations of nisin (1–400 µg/ml), ampicillin (0.1–16 µg/ml), EDTA (1 mM–100 mM), ceftriaxone (0.1–16 µg/ml) and cefotaxime (0.1–16 µg/ml). Overnight growth was monitored by measuring the optical density at 620 nm.

#### (B) Micro broth dilution assay

Various antimicrobial agents (nisin, antibiotics and EDTA) were added to the bacterial suspension containing approximately 10^7^ CFU/ml of log phase cells of the bacterial strain in a 96-well flat bottom plate. Plates were incubated at 37°C for 18–24 hours. Inhibition of bacterial growth was determined by comparing the change in turbidity at OD_600_ in the presence of agent to that in the absence of agent.

### Fractional inhibitory concentrations (FIC)

To evaluate synergy, the fractional inhibitory concentration (FIC) index was determined by checkerboard microtitre test, using an 8-by-8 well configuration at 64 different combinations as described earlier [7], [22]. Briefly, twofold serial dilutions of each antibiotic and nisin were prepared, with concentrations ranging from 0.016 to 2 times the MIC. Ten microliters of each nisin dilution was added to the wells of a 96-well plate in a vertical orientation, and same amount of each antibiotic dilution was added in a horizontal orientation so that the plates contained various combinations of the two agents (Illustrated in Data S1). Each well was then supplemented with 80 µ (10^7^ CFU/ml) of serovar Typhimurium, and the plate was incubated at 37°C. Wells not containing any antibacterial agent were used as positive growth controls.

The FIC was calculated after dividing the MIC of the tested agent in combination by the MIC of tested agent alone separately. The FIC index, obtained by adding both the FICs, was interpreted as indicating a synergistic effect when it was ≤0.5, as additive or indifferent when it was >0.5 and ≤2.0, and as antagonistic when it was >2.0.

### Time-kill Assay

The *in-vitro* synergy as estimated by checkerboard technique was further confirmed by time-kill assay. The antimicrobial action of all the antibacterial agents (nisin, EDTA and antibiotics) was determined at their respective MICs, when the agents were used alone or in combination. Briefly, all the agents (alone and in combination) were added to 3 ml of nutrient broth containing 10^7^ CFU of serovar Typhimurium and then incubated at 37°C. For each agent, six flasks of nutrient broth were prepared corresponding to various time intervals i.e. 0 hour, 3 hour, 6 hour, 12 hour, 24 hour, and 48 hour. For all the agents, at the given time intervals, entire sample (3 ml) divided into 30 aliquots of 100 µl each was plated on to 30 MacConkey agar plates. The plates were incubated at 37°C for 24 hours for enumeration of colony forming units.

### Scanning electron microscopy

The ultrastructural changes if any, induced by the combinations were studied by scanning electron microscopy (SEM). For the SEM study, washed and concentrated cells of serovar Typhimurium from the log phase were incubated with various antimicrobial agents alone at their sub-inhibitory concentrations [at 0.25 times the MIC i.e. nisin (100 µg/ml), ampicillin (0.25 µg/ml), ceftriaxone (3 µg/ml), cefotaxime (2 µg/ml), EDTA (7.5 mM)] and in combinations at their MICs [i.e. nisin (100 µg/ml) + ampicillin (0.5 µg/ml), nisin (4 µg/ml) + ceftriaxone (0.75 µg/ml), and nisin (5 µg/ml) + cefotaxime (1 µg/ml) and nisin (20 µg/ml) + EDTA (20 mM)] for 2 hours at 37°C. Using agents alone at their MICs, the damage observed was too extensive to demonstrate the changes on the bacterial surface. Therefore, the above mentioned concentrations were selected after standardization where the action of the agent(s) was best appreciated. Treated samples were centrifuged (800 rpm for 20 mins); pellets were resuspended and fixed in 2% glutaraldehyde (1 hour at room temperature), dehydrated in a series of graded alcohol baths, transferred on to glass cover slip and then subjected to air drying. Finally, the cover slips containing samples were mounted on aluminium stubs, coated with gold-palladium at a thickness of 200A°, and examined for the change in morphology by scanning electron microscope (Hitachi S-3400N model).

### *In-vivo* study using various combinations against murine *Salmonella* infection

**Figure 1:**
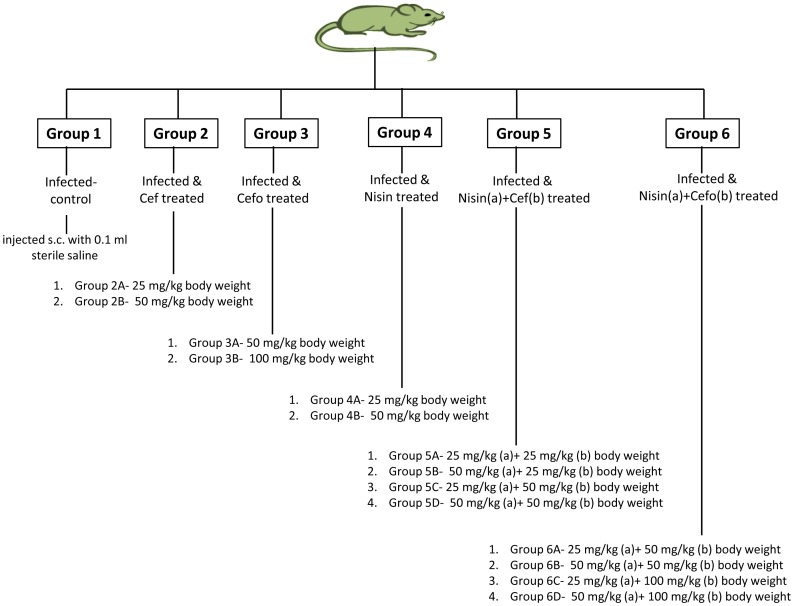
Diagrammatic representation of various treatment groups made for *in-vivo* studies. Uninfected-control group comprising 6 mice was also used in the study (not shown).

The magnitude of *in-vitro* synergism observed between nisin-ceftriaxone and nisin-cefotaxime prompted us to further assess their clinical relevancy through *in-vivo* study using a murine model. Ninety mice were infected with 0.25 ml of 10^7^ CFU of serovar Typhimurium orally. Seven days after the challenge, establishment of *Salmonella* infection was confirmed by bacterial translocation into the intestines, livers, and spleens of the infected mice. At 7 days post-infection, mice were divided into 6 groups each comprising of at-least 6 mice. A group of six mice was set aside which served as uninfected-control. Various treatment groups have been represented in figure 1. All the test agents were injected in four doses subcutaneously (s.c.) after 12 hour interval individually and in combination. The doses used were selected on the basis of pilot studies. At 48 hour post-therapy, mice were sacrificed and their livers, small intestines, and spleens were removed aseptically, rinsed in isotonic saline solution and weighed. Ten percent (w/v) of tissue homogenates were prepared in sterile PBS using a Potter Elvehjem homogenizer. Serial 10-fold dilutions of each homogenate were plated on MacConkey agar medium for enumeration of CFU per organ after incubation at 37°C for 24 hours.

Another set of experiment was performed to evaluate the survival assay wherein, thirty mice were infected with 0.25 ml of 10^7^ CFU of serovar Typhimurium orally. At 7 days post-infection, mice were divided into 3 groups each comprising of 10 mice. Group 1 was injected subcutaneously with sterile saline. Group 2 was injected with four doses of nisin (50 mg/kg body weight) + ceftriaxone (50 mg/kg body weight) subcutaneously (s.c.) after 12 hour interval. Group 3 was injected with four doses of nisin (50 mg/kg body weight) + cefotaxime (100 mg/kg body weight) subcutaneously (s.c.) each after 12 hour interval. The numbers of surviving mice were recorded at 12 hour intervals over 21 days post-infection.

### Statistical analysis

Data were expressed as means ± standard deviations for three to five independent experiments. Statistical analysis was done by Student's unpaired *t* test and one-way analysis of variance (ANOVA), followed by pairwise comparison procedures (Tukey test), using Jandel Sigma Stat statistical software, version 2.0. In all cases, statistical significance was defined as having a *P* value of <0.05.

## Results

### Radial diffusion assay

No zone of inhibition was observed when nisin was used alone. Agar well diffusion assay indicated that nisin-EDTA, nisin-ampicillin, nisin-ceftriaxone and nisin-cefotaxime acted in conjunction for serovar Typhimurium which was evidenced by the increase in zone of growth inhibition when used together as compared to the size of zone when these agents were used alone. However, no difference in zone of inhibition was observed when nisin-chloramphenicol and nisin-ciprofloxacin were used together (Data S2).

### MIC determination

MIC for various agents was determined and confirmed by both macro and micro broth dilution assay. For ampicillin, ceftriaxone, cefotaxime and EDTA, MIC was observed to be 1 µg/ml, 12 µg/ml, 8 µ/ml, and 30 mM, respectively. Using nisin, no inhibition of growth was observed till 400 µg/ml (Table 1). According to Clinical and Laboratory Standards Institute guidelines (CLSI guidelines) [23], serovar Typhimurium was classified as resistant to ceftriaxone and cefotaxime, and susceptible to ampicillin [as per the MIC breakpoints for ceftriaxone and cefotaxime susceptible (≤1), intermediate (1–4) and resistant (≥4), for ampicillin susceptible (≤8), intermediate (8–32) and resistant (≥32)].

**Table 1:** MIC values and FIC index of various antimicrobial agents alone and in combinations against *Salmonella enterica* serovar Typhimurium as determined by broth dilution technique and checkerboard method.

Antimicrobial agents	Conc. Used (µg/ml)	MIC (µg/ml)	MIC in combination		FIC		FIC Index
			Nisin (µg/ml))	Agents (µg/ml)	Nisin*	Agents	
Ampicillin	0.1–16	1	100	0.5	0.25	0.5	0.75
Ceftriaxone	0.1–32	12	4	0.75	0.01	0.063	0.073
Cefotaxime	0.1–32	8	5	1	0.0125	0.125	0.138
EDTA	5–100 mM	30 mM	20	20 mM	0.05	0.66	0.71
Nisin	1–400	Till 400 no MIC	—	—	—	—	—

* - MIC of nisin was considered as 400 (µg/ml)

### Fractional inhibitory concentrations (FIC)

The combination of nisin-ceftriaxone and nisin-cefotaxime were found to be highly synergistic as indicated by FIC indices, which were ≤0.5 for serovar Typhimurium. However, nisin-ampicillin and nisin-EDTA combinations were found to have additive effects against serovar Typhimurium as indicated by FIC indices, which were 0.75 and 0.71 respectively (Table 1).

### Time-kill Assay

**Figure 2:**
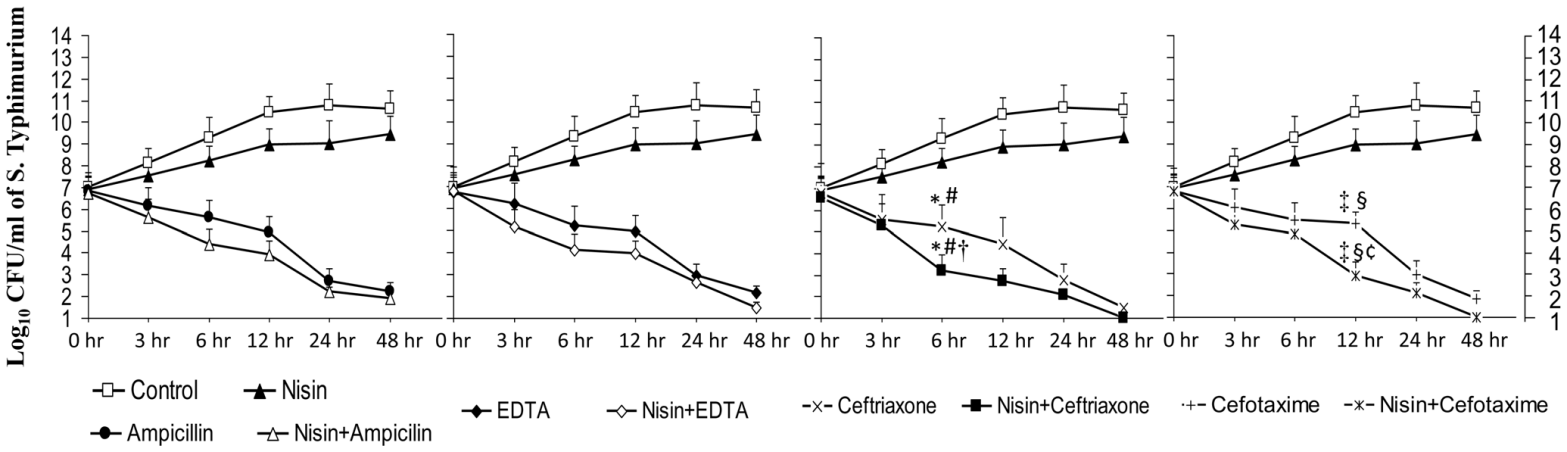
Log_10_ CFU/ml of *Salmonella enterica* serovar Typhimurium at different time intervals in presence of antibiotic alone (MIC) and in presence of nisin + antibiotic (1×MIC +1×MIC). Values are expressed as mean ± standard deviation of three individual values. *, P <0.001 versus log_10_ CFU of serovar Typhimurium after 6 hour in the absence of any antibacterial agent (control); #, P<0.01 versus log_10_ CFU of serovar Typhimurium after 6 hour in the presence of nisin (400 µg/ml); †, P<0.05 versus log_10_ CFU of serovar Typhimurium after 6 hour in the presence of ceftriaxone (12 µg/ml); ‡, P<0.001 versus log_10_ CFU of serovar Typhimurium after 12 hour in the absence of any antibacterial agent (control); §, P<0.01 versus log_10_ CFU of serovar Typhimurium after 12 hour in the presence of nisin (400 µg/ml); ¢, P<0.05 versus log_10_ CFU of serovar Typhimurium after 12 hour in the presence of cefotaxime (8 µg/ml).

All synergistic interactions inferred from checkerboard analysis were reassessed by time-kill assay performed with nisin in combination with ampicillin, ceftriaxone, cefotaxime and EDTA. Only nisin-ceftriaxone and nisin-cefotaxime combination showed synergy after 6 hours and 12 hours respectively which was evident by a ≥2 log_10_ unit reduction as compared to the killing of same magnitude by each agent alone, which seems to occur at 24 hours (approximately) indicating that the combinations could kill bacteria more rapidly than each single agent against serovar Typhimurium (Figure 2). Using nisin-ceftriaxone and nisin-cefotaxime combination, few colonies were observed when plated from the flasks after 24 hour incubation. However, when the incubation was prolonged upto 48 hours, interestingly no colony was observed.

### Scanning electron microscopy

**Figure 3:**
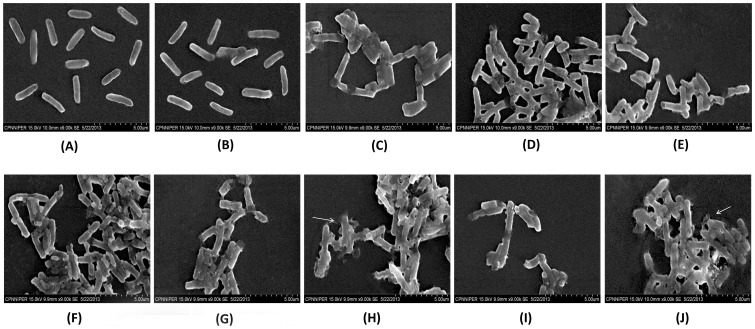
Scanning electron micrographs of *Salmonella enterica* serovar Typhimurium after 2 hours in presence of (A) normal saline (B) nisin (100 µg/ml) (C) EDTA (7.5 mM) (D) nisin (20 µg/ml) +EDTA (20 mM) (E) ampicillin (0.25 µg/ml) (F) nisin (100 µg/ml) + ampicillin (0.5 µg/ml) (G) ceftriaxone (3 µg/ml) (H) nisin (4 µg/ml) + ceftriaxone (0.75 µg/ml) (I) cefotaxime (2 µg/ml) (J) nisin (5 µg/ml) + cefotaxime (1 µg/ml).

In contrast to control samples, marked changes were evident on the outer membranes of serovar Typhimurium treated with various combinations. However, nisin-ceftriaxone and nisin-cefotaxime combinations seem to cause extensive damage to the bacterial cells. It is indicated that both these combinations could lead to clubbing and disruption of bacterial membrane after 2 hours of treatment period (Figure 3).

### Therapeutic potential and synergistic efficacy of nisin and antibiotics against experimental salmonellosis

Therapeutic efficacy of antimicrobial agents alone and in conjunction was investigated in terms of reduction in the number of salmonellae in different target organs of mice infected with serovar Typhimurium after 48 hours of first dose of chemotherapy.

**Figure 4:**
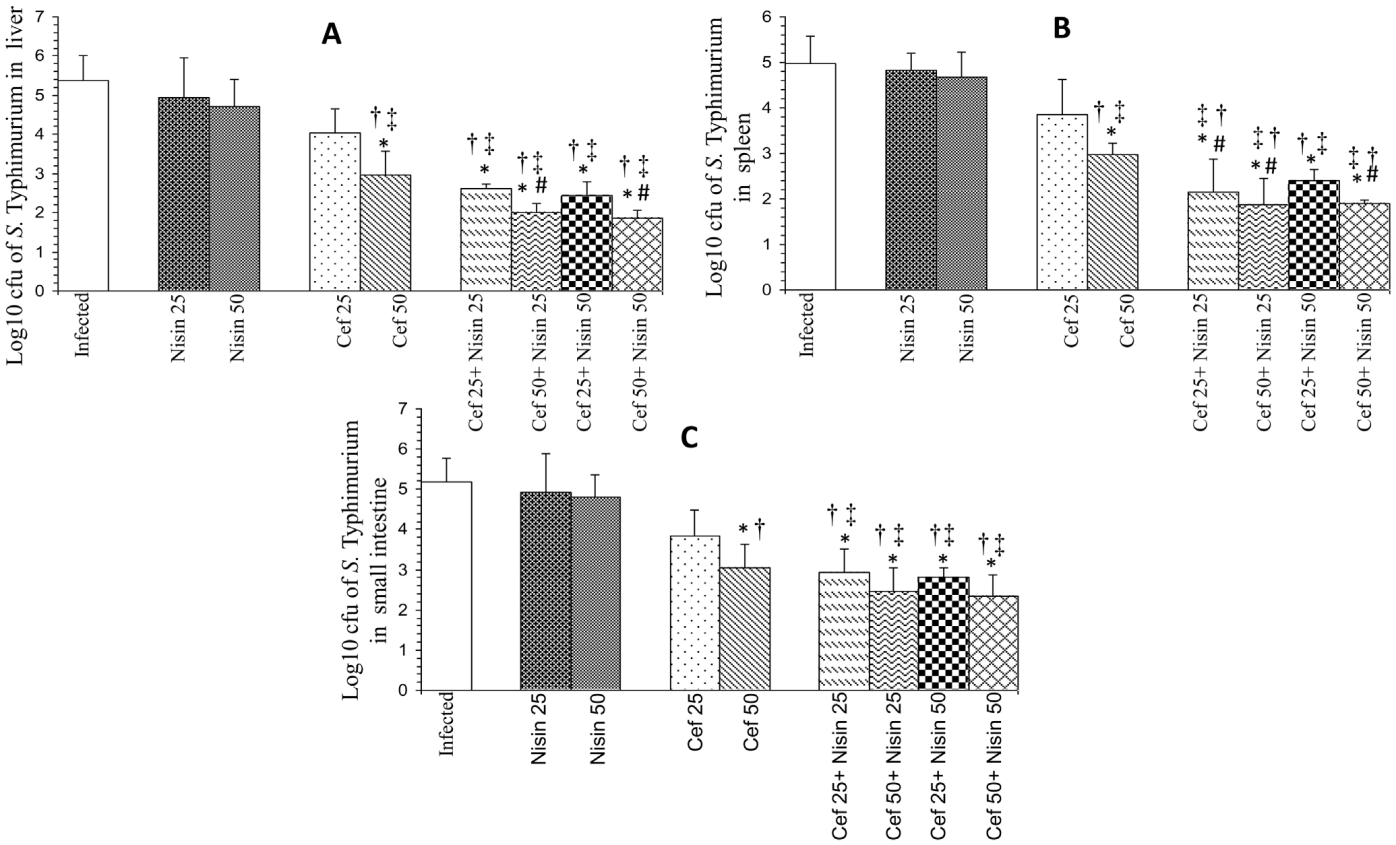
Log_10_ CFU of *Salmonella enterica* serovar Typhimurium in (A) livers, (B) spleens and (C) small intestines of infected mice after 48 hours of therapy with nisin and ceftriaxone (cef) separately and in combination. Values are expressed as mean ± S.D. of three independent experiments. *, P<0.05 versus log_10_ CFU of serovar Typhimurium in untreated mice (control); #, P<0.05 versus log_10_ CFU of serovar Typhimurium in mice treated with Cef (25 mg/kg body weight); †, P<0.05 versus log_10_ CFU of serovar Typhimurium in mice treated with nisin (25 mg/kg body weight); ‡, P<0.05 versus log_10_ CFU of serovar Typhimurium in mice treated with nisin (50 mg/kg body weight). Uninfected-control group comprising 6 mice was also used in the study (not shown as no bacterial load was recovered from this group).

**Figure 5:**
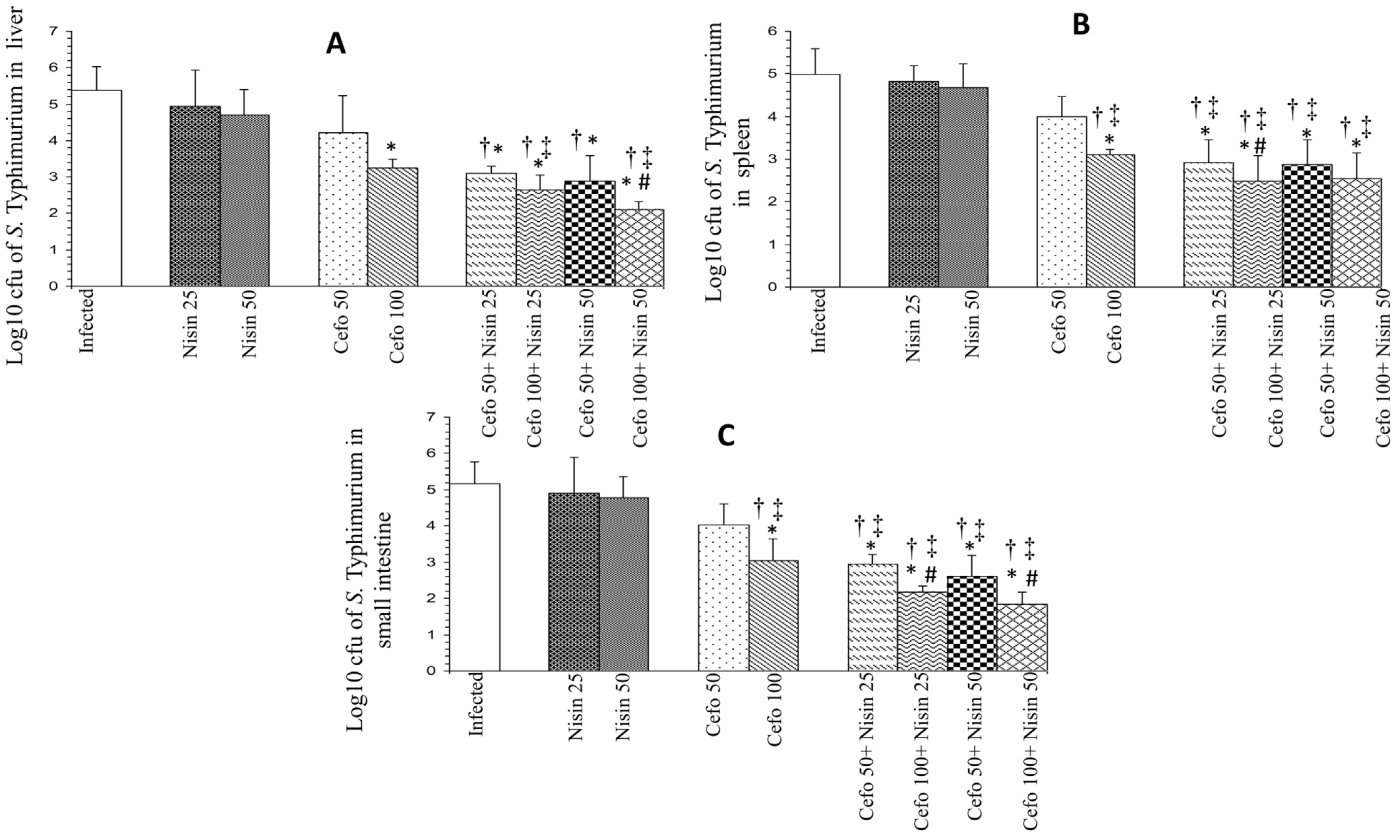
Log_10_ CFU of *Salmonella enterica* serovar Typhimurium in (A) livers, (B) spleens and (C) small intestines of infected mice after 48 hours of therapy with nisin and cefotaxime (cefo) separately and in combination. Values are expressed as mean ± S.D. of three independent experiments. *, P<0.05 versus log10 CFU of serovar Typhimurium in untreated mice (control); #, P<0.05 versus log_10_ CFU of serovar Typhimurium in mice treated with Cefo (50 mg/kg body weight); †, P<0.05 versus log_10_ CFU of serovar Typhimurium in mice treated with nisin (25 mg/kg body weight); ‡, P<0.05 versus log_10_ CFU of serovar Typhimurium in mice treated with nisin (50 mg/kg body weight). Uninfected-control group comprising 6 mice was also used in the study (not shown as no bacterial load was recovered from this group).

In the livers of treated mice, nisin alone did not show any significant effect as 0.42 and 0.67 log-unit decrease was observed after treatment with 25 and 50 mg nisin/kg body weight, while log unit decreases in bacterial loads were 1.34 and 2.42 for 25 and 50 mg **cef**/kg body weight (Figure 4A) and 1.15 and 2.13 for 50 and 100 mg **cefo**/kg body weight, respectively (Figure 5A). Co-administration of 25 mg nisin/kg body weight with 25 and 50 mg **cef**/kg body weight gave higher log unit decreases of 2.75 and 3.36, respectively, while 2.94 and 3.5 log-unit decrease was observed after co-administration of 50 mg nisin/kg body weight with 25 and 50 mg **cef**/kg body weight (Figure 4A). Likewise, co-administration of 25 mg nisin/kg body weight with 50 and 100 mg **cefo**/kg body weight gave higher log unit decreases of 2.27 and 2.72, respectively, while 2.49 and 3.26 log-unit decrease was observed after co-administration of 50 mg nisin/kg body weight with 25 and 50 mg **cefo**/kg body weight (Figure 5A).

In spleens of mice, log unit decreases in bacterial loads were observed to be 1.13, 2.02, 0.99, and 1.88 after treatment with 25 and 50 mg **cef**/kg body weight and with 50 and 100 mg **cefo**/kg body weight, respectively. Treatment with nisin alone gave 0.16 and 0.31 log-unit decrease respectively. In this case also, the adjunct therapy of nisin (25 and 50 mg/kg body weight) with 25 and 50 mg **cef**/kg body weight was found to be more effective, as higher log unit decreases of 2.83, 3.11, 2.6 and 3.10, respectively, were observed (Figure 4B). Similarly, the adjunct therapy of nisin (25 and 50 mg/kg body weight) with 50 and 100 mg **cefo**/kg body weight showed 2.06, 2.49, 2.11 and 2.44 log unit decrease, respectively (Figure 5B).

In the small intestines, **cef** (25 and 50 mg/kg body weight) as well as **cefo**, when used alone (50 and 100 mg/kg body weight) gave a decrease in bacterial loads of 1.33, 2.13, 1.13 and 2.12 log units compared to untreated-controls, respectively whereas there were log unit decreases of 0.26 and 0.38 in the number of salmonellae in small intestines of mice treated with 25 and 50 mg nisin/kg body weight, respectively. However, when 25 and 50 mg of nisin/kg body weight were used in combination with **cef** (25 and 50 mg/kg body weight), the log unit decreases in intestinal bacterial loads were found to be 2.24, 2.71, 2.35 and 2.83 units, respectively (Figure 4C). Similar trend was observed using nisin-cefotaxime combination. Combination of nisin (25 and 50 mg/kg body weight) with **cefo** (50 and 100 mg/kg body weight) showed 2.23, 3, 2.57 and 3.34 log-unit decrease respectively (Figure 5C).

In other words, bacterial loads recovered from all target organs, when treated with lower doses of each agent in conjunction were observed to be at par with the recovered bacterial loads observed when the agents were used alone at twofold higher concentrations.

In the survival assay, mice from both the groups receiving treatments showed an increase survival as compared to that in the infected-control group. In the infected-control group 50%, 70% and 100% mortality was observed at 9, 11 and 14 days post-infection respectively. However, 90% and 80% survival was observed up to 21 days in nisin-ceftriaxone and nisin-cefotaxime treated groups respectively (Data S3). 10 and 20% mortality observed in the treated groups may be due to some other reason including their immuno-compromised state. However, further investigation such as histology of the liver and intestinal tissues along with blood evaluations would substantiate the clarity of the survival curve.

## Discussion

Synergy between two antimicrobial drugs is obtained when the combination of drugs elicits a more than additive effect as opposed to each drug alone. Its clinical importance results in taking advantage of different mechanisms of action of the agents involved [24] and providing an additional therapeutic choice in difficult-to-treat infections [25]. A major problem in combating *Salmonella* is its outer membrane (OM). The OM is a lipid bilayer in which the predominant lipid is the lipopolysaccharide (LPS). The LPS layer forms a tight shield [26] and acts as a barrier to many compounds, including antibiotics, hydrophobic compounds, detergents and dyes [27]. The anionic LPS layer is stabilized by divalent cations. If these cations are removed, lipopolysaccharide molecules are released from the OM, exposing the underlying phospholipid bilayer and jeopardizing the integrity of the OM [27]. Bacteriocins have been found to have many distinct mechanisms of action that differ from those of antibiotics. These mechanisms can be broadly divided into those that function primarily at the cell envelope and those that are active primarily within the cell, affecting gene expression and protein production [28]. Nisin has been reported to exert its antimicrobial activity through both pore formation in the membrane and by binding to lipid II, which is an essential intermediate in peptidoglycan biogenesis. Precisely how this occurs and whether a receptor is involved is yet to be elucidated [28], [29]. However, nisin alone is generally not effective against Gram-negative bacteria due to the barrier provided by their outer membranes.

In the present study, only β-lactam antibiotics i.e. ampicillin, ceftriaxone and cefotaxime could confer higher inhibitory activity when used in combination with nisin. Owing to the properties of β-lactam antibiotics as metal chelators as suggested by Ji *et al* [30], it may be possible that these antibiotics lead to changes in cell morphology that might have helped in increasing the permeabilization of nisin across bacterial OM, enabling it to form pores in inner membrane. Additionally, this might allow more influx of the antibiotics resulting in more inhibition and hence, the increased zone size and altered morphology. These findings are in concordance with the earlier reports showing such type of changes using β-lactam antibiotics [31], [32]. Further, Nisin may also render the drug efflux pumps ineffective, thereby increasing cellular drug accumulation by altering cell permeability, leading to collapse of membrane potential and causing rapid efflux of materials present in cytosolic milieu [33], [34]. These mechanisms might have acted mutually leading to enhanced anti-*Salmonella* effect as observed by FIC index and time-kill assay in the present study. In order to check any emergence of resistance in the surviving bacterial population, organism surviving after 24 hour incubation in time-kill assay were again grown and retreated with the same combinations. The combinations were still found to be effective. In view of the fact that the SEM image relies on surface processes rather than transmission, it is able to image large area (many cells) and has a great depth of field, and so can produce images that are reasonably good representations of the three-dimensional shape of the sample. Therefore, the morphological alterations were evaluated by SEM. However, in continuation to scanning electron microscopy, TEM analysis has been planned for future studies which might give more detailed and meaningful information regarding the mechanism of action.Despite being a β-lactam, a less effective show displayed by ampicillin can be explained by the presence of ampicillin inactivating enzyme such as β-lactamase, thereby making this very combination ineffective. However, β-lactamases are not effective against higher generation cephalosporins with an oxyimino side chain, such as cefotaxime, ceftazidime, ceftriaxone or cefepime [35]. It may also be inferred from the results that nisin has the potential to augment the activity of the antibiotics at much lower MICs thereby rendering serovar Typhimurium sensitive to ceftriaxone and cefotaxime, which was earlier observed to be resistant as per the CLSI guidelines.

The results of *in-vivo* studies also indicated synergism between nisin-ceftriaxone and nisin-cefotaxime, as more clearance of salmonellae from the target organs was observed using the combination. These results are in concordance with earlier reports suggesting a synergistic effect of antimicrobial peptides with β-lactam antibiotics [7], [36], [37]. The most interesting finding of our study was that when nisin was used in conjunction with ceftriaxone and cefotaxime, the log unit decreases in the numbers of salmonellae in all target organs were observed to be comparable to the decreases observed when the agents (ceftriaxone and cefotaxime) were used alone at twofold higher concentrations. Therefore, it reduced the therapeutic dosage of both agents to half (from 50 to 25 mg/kg of ceftriaxone and from 100 to 50 mg/kg of cefotaxime) while maintaining the increased therapeutic efficacy. Moreover, treatment with the combination also enhanced the survival rate of the infected animals. However, it is worth mentioning here that though the treatment resulted in significant reduction in the bacterial load but the organisms could still be isolated from the organs indicating that animals have not fully rejuvenated. Had the treatment been either started earlier or prolonged, absolute recovery would have been possible.

Thus judicious and restricted antibiotic use might help in withdrawal of selective pressure, thereby reducing the chances of developing resistance induced by the action of extended spectrum β-lactamases (ESBL) [38]. Therefore, the present investigation can now be used as a blueprint for the development of new class of highly efficient antibiotics for the treatment of Gram-negative infections.

In conclusion, nisin-ceftriaxone and nisin-cefotaxime combinations demonstrated excellent *in-vitro* and *in-vivo* synergism against the serovar Typhimurium. This study highlights that nisin has the potential to act in conjunction with conventional antibiotics at much lower concentrations (as evidenced from *in-vitro* and *in-vivo* results). Thus, in addition to having the faster effect using the combination, reducing the concentration of the antibiotics may reduce the chances of developing resistance in the pathogens besides reducing the associated side effects of the former. Nisin-antibiotic combination appears to fulfill the dual purpose over nisin-EDTA combination as i) the former may reduce the effective therapeutic dose of the antibiotics and ii) would avoid the otherwise chelating property of the latter in the *in-vivo* situation. Given the value addition potential of nisin, its combination with antibiotics can thus be exploited instead of nisin-EDTA combination both against *Salmonella* and other Gram-negative infections as well. However, the effect of these combinations against the intestinal microbiota needs to be explored in future studies in order to consider the combinations clinically more relevant.

## Supporting Information

Data S1 **An illustration of checkerboard assay showing 64 combinations used to determine FIC.**
(DOC)

Data S2 **Zone of inhibition in presence of various antimicrobial agents.**
(DOC)

Data S3 **Survival analysis of BALB/c mice infected with** ***Salmonella enterica*** **serovar Typhimurium followed by treatment with nisin (50 mg/kg body weight) + ceftriaxone (50 mg/kg body weight) and nisin (50 mg/kg body weight) + cefotaxime (100 mg/kg body weight) subcutaneously (s.c.).**
(TIF)
