# Supplementary material for: Value Addition in the Efficacy of Conventional Antibiotics by Nisin against Salmonella
Source: PLoS One. 2013 Oct 8;8(10):e76844. doi: 10.1371/journal.pone.0076844 (PMC3792866; doi:10.1371/journal.pone.0076844)
Supplement: Data S1 — An illustration of checkerboard assay showing 64 combinations used to determine FIC. (DOC) [file pone.0076844.s001.doc]

**Data S1:** An illustration of checkerboard assay showing 64 combinations used to determine FIC.

|  | **Agent A** | | | | | | | |
| --- | --- | --- | --- | --- | --- | --- | --- | --- |
| **Agent B** | 0.016MIC (A)  0.016MIC(B) | 0.032MIC(A)  0.016MIC(B) | 0.063 MIC(A)  0.016MIC(B) | 0.125 MIC(A)  0.016MIC(B) | 0.25 MIC(A)  0.016MIC(B) | 0.5 MIC(A)  0.016MIC(B) | MIC(A)  0.016MIC(B) | 2 MIC(A)  0.016MIC(B) |
|  | 0.016MIC(A)  0.032MIC(B) | 0.032 MIC(A)  0.032MIC(B) | 0.063 MIC(A)  0.032MIC(B) | 0.125 MIC(A)  0.032MIC(B) | 0.25 MIC(A)  0.032MIC(B) | 0.5 MIC(A)  0.032MIC(B) | MIC(A)  0.032MIC(B) | 2 MIC(A)  0.032MIC(B) |
|  | 0.016MIC(A)  0.063MIC(B) | 0.032 MIC(A)  0.063MIC(B) | 0.063 MIC(A)  0.063MIC(B) | 0.125 MIC(A)  0.063MIC(B) | 0.25 MIC(A)  0.063MIC(B) | 0.5 MIC(A)  0.063MIC(B) | MIC(A)  0.063MIC(B) | 2 MIC(A)  0.063MIC(B) |
|  | 0.016MIC(A)  0.125MIC(B) | 0.032 MIC(A)  0.125MIC(B) | 0.063 MIC(A)  0.125MIC(B) | 0.125 MIC(A)  0.125MIC(B) | 0.25 MIC(A)  0.125MIC(B) | 0.5 MIC(A)  0.125MIC(B) | MIC(A)  0.125MIC(B) | 2 MIC(A)  0.125MIC(B) |
|  | 0.016MIC(A)  0.25MIC(B) | 0.032 MIC(A)  0.25MIC(B) | 0.063 MIC(A)  0.25MIC(B) | 0.125 MIC(A)  0.25MIC(B) | 0.25 MIC(A)  0.25MIC(B) | 0.5 MIC(A)  0.25MIC(B) | MIC(A)  0.25MIC(B) | 2 MIC(A)  0.25MIC(B) |
|  | 0.016MIC(A)  0.5MIC(B) | 0.032 MIC(A)  0.5MIC(B) | 0.063 MIC(A)  0.5MIC(B) | 0.125 MIC(A)  0.5MIC(B) | 0.25 MIC(A)  0.5MIC(B) | 0.5 MIC(A)  0.5MIC(B) | MIC(A)  0.5MIC(B) | 2 MIC(A)  0.5MIC(B) |
|  | 0.016MIC(A)  MIC(B) | 0.032 MIC(A)  MIC(B) | 0.063 MIC(A)  MIC(B) | 0.125 MIC(A)  MIC(B) | 0.25 MIC(A)  MIC(B) | 0.5 MIC(A)  MIC(B) | MIC(A)  MIC(B) | 2 MIC(A)  MIC(B) |
|  | 0.016MIC(A)  2 MIC(B) | 0.032 MIC(A)  2 MIC(B) | 0.063 MIC(A)  2 MIC(B) | 0.125 MIC(A)  2 MIC(B) | 0.25 MIC(A)  2 MIC(B) | 0.5 MIC(A)  2 MIC(B) | MIC(A)  2 MIC(B) | 2 MIC(A)  2 MIC(B) |
