# Supplementary material for: Value Addition in the Efficacy of Conventional Antibiotics by Nisin against Salmonella
Source: PLoS One. 2013 Oct 8;8(10):e76844. doi: 10.1371/journal.pone.0076844 (PMC3792866; doi:10.1371/journal.pone.0076844)
Supplement: Data S2 — Zone of inhibition in presence of various antimicrobial agents. (DOC) [file pone.0076844.s002.doc]

**Data S2: Zone of inhibition in presence of various antimicrobial agents**

| **Agent** | **Zone size (mm)** |
| --- | --- |
|  | ***S.* Typhimurium** |
| Nisin | No zone |
| EDTA | 10 |
| EDTA+Nisin | 21 |
| Ampicillin | 20 |
| Ampicillin+Nisin | 24 |
| Chloramphenicol | 8 |
| Chloramphenicol+Nisin | 8 |
| Ciprofloxacin | 28 |
| Ciprofloxacin+Nisin | 28 |
| Ceftriaxone | 19 |
| Ceftriaxone+Nisin | 28 |
| Cefoaxime | 20 |
| Cefoaxime+Nisin | 28 |
