# Supplementary figures and images for: Value Addition in the Efficacy of Conventional Antibiotics by Nisin against Salmonella
Source: PLoS One. 2013 Oct 8;8(10):e76844. doi: 10.1371/journal.pone.0076844 (PMC3792866; doi:10.1371/journal.pone.0076844)

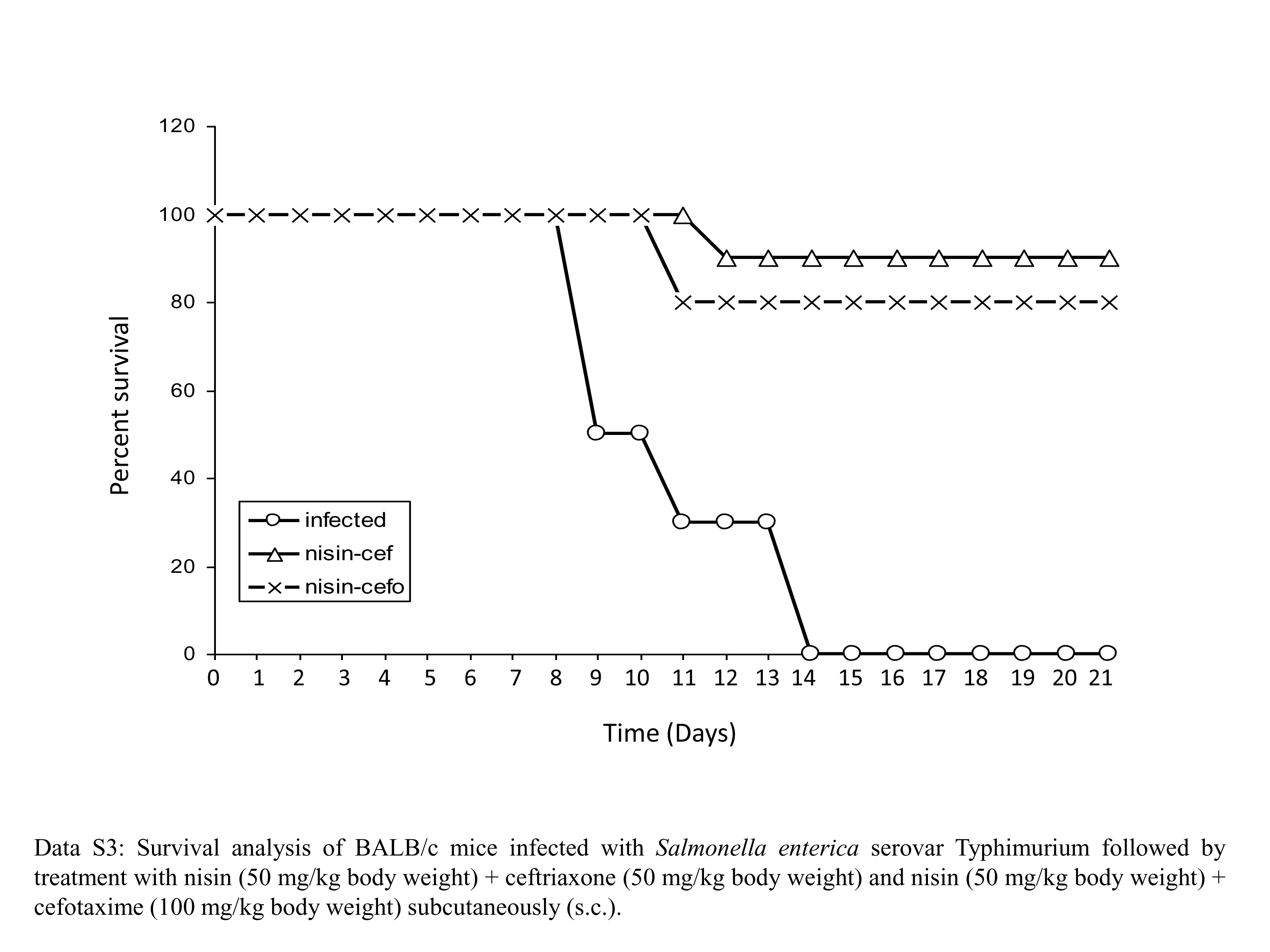

Supplement: Data S3 — Survival analysis of BALB/c mice infected with Salmonella enterica serovar Typhimurium followed by treatment with nisin (50 mg/kg body weight) + ceftriaxone (50 mg/kg body weight) and nisin (50 mg/kg body weight) + cefotaxime (100 mg/kg body weight) subcutaneously (s.c.). (TIF) [file pone.0076844.s003.tif]
